# Improved image quality and reduced acquisition time in prostate T2-weighted spin-echo MRI using a modified PI-RADS-adherent sequence

**DOI:** 10.1186/s41747-025-00595-w

**Published:** 2025-05-24

**Authors:** Stephen J. Riederer, Eric A. Borisch, Adam T. Froemming, Roger C. Grimm, Sara Hassanzadeh, Akira Kawashima, Naoki Takahashi, John Thomas

**Affiliations:** 1 Department of Radiology, Mayo Clinic, Rochester, MN USA; 2 Department of Radiology, Mayo Clinic, Phoenix, AZ USA

**Keywords:** Magnetic resonance imaging, Prostate, Prostatic neoplasms, PI-RADS, Signal-to-noise ratio

## Abstract

**Background:**

Prostate imaging reporting and data system (PI-RADS) v2.1 guidelines for magnetic resonance imaging acquisition define a standard of 0.40 mm × 0.70 mm in-plane resolution (0.280 mm^2^ pixel area), but adherence has been challenging. We questioned if a modification of a PI-RADS-adherent T2-weighted (T2WI) sequence to one having equivalent pixel area could allow reduced acquisition time but provide improved diagnostic quality (DQ).

**Methods:**

An adherent T2WI sequence was modified by reducing the frequency sampling, thereby reducing the signal bandwidth (BW). This was compensated by increasing the phase sampling for an equivalent pixel area (0.50 mm × 0.57 mm = 0.285 mm^2^). The BW reduction allowed a two-fold reduction in averaging, also enabling reduced acquisition time. The adherent and modified sequences were evaluated in phantoms and 62 consecutive prostate MRI subjects. Images were evaluated individually by four radiologists using a four-point DQ scale and using prostate imaging quality (PI-QUAL)v2. Each reviewer also indicated any sequence preference. The Wilcoxon test was used.

**Results:**

In the phantom, mean signal-to-noise ratios were equivalent for the two sequences; superior frequency resolution for the adherent sequence, and superior phase resolution for the modified sequence were shown. Across 62 participants, the median acquisition time was reduced by 23%, from 3:55 min:s to 3:01 min:s. For all three means of comparison (DQ, PI-QUALv2, reader preference), the modified sequence was significantly superior (*p* ≤ 0.037).

**Conclusion:**

Modification of the PI-RADS standard (0.40-mm frequency resolution) to an equivalent, more isotropic pixel area (0.28 mm^2^) reduced acquisition time and improved image quality.

**Relevance statement:**

Generalization of the PI-RADSv.2.1 minimum technical standard for T2WI in-plane resolution to be more isotropic preserves the targeted high resolution, allowing reduced acquisition time, also reducing motion sensitivity, and improving image quality. This approach may also reduce the need for rescanning poor-quality sequences.

**Key Points:**

- PI-RADSv2.1 suggests a standard T2WI sequence with 0.40 × 0.70 mm^2^ in-plane resolution.
- A modified PI-RADSv.2.1-adherent T2WI sequence with equivalent but more isotropic pixel area (0.50 × 0.57 mm^2^) allowed reduced scan times by 23% and significantly improved DQ.
- Superiority of the modified sequence appears due to reduced motion sensitivity.

**Graphical Abstract:**

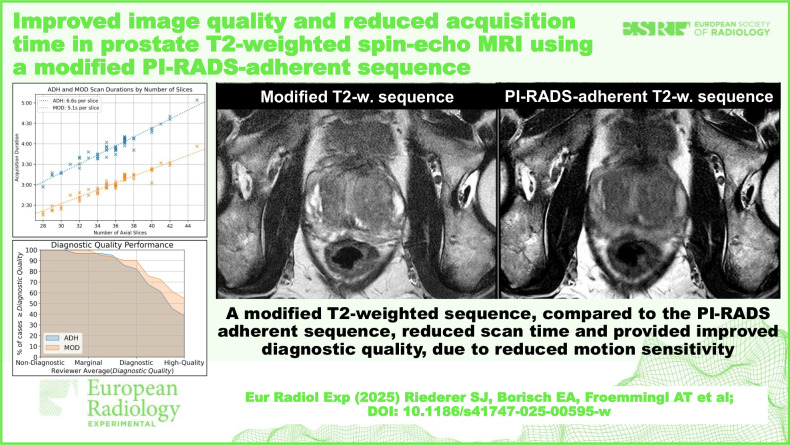

## Background

The prostate imaging and reporting data system (PI-RADS) was created in 2012 [1] to provide guidance in the acquisition and interpretation of magnetic resonance imaging (MRI) of biopsy-naïve men suspected of prostate cancer. PI-RADS has subsequently undergone revisions to version 2 [2] and the current version 2.1 [3]. This has been useful in the standardization of prostate MRI interpretation [4–6] and for consistent data collection in multiinstitutional studies [7, 8]. PI-RADS provides minimum technical standards for acquisition parameters for the three underlying sequences: spin-echo T2-weighted imaging (T2WI); echo-planar diffusion-weighted imaging; and gradient-echo dynamic contrast-enhanced study. However, depending upon local implementation, individual sites adhere to these to a greater or lesser degree. A 2018 review of 107 sites showed that of the multiple PI-RADSv2 parameters, adherence to the 0.40 mm frequency resolution for T2WI was lowest, only 16.8% [9]. A 2019 review [10] showed similar low adherence, 15%. Another review of 62 prostate MRI exams referred for secondary interpretation found only 52% adhered to the 0.40-mm frequency resolution standard for T2WI [11]. Minimum technical standards for T2WI in-plane resolution did not change from PI-RADSv2 to PI-RADSv2.1. More recently, in preparation for the multiinstitutional “Prostate Imaging Using MRI ± contrast enhancement”−PRIME trial, the organizers prospectively evaluated acquisition protocols used on the participating MRI scanners [12]. Twenty-one of 64 sites (33%) did not initially adhere to the 0.40 × 0.70 mm^2^ in-plane spatial resolution. Although 19 of these 21 were corrected after feedback, the original statistic clearly shows a general lack of adherence to this technical standard.

In addition to documenting the low adherence, Sackett et al [11] showed limited correlation of reader-assessed image quality to PI-RADS adherence: adherence to PI-RADS minimum technical standards did not guarantee good results, while workarounds sometimes did. This and the other already mentioned experiences [9, 10] questioned whether PI-RADS minimum technical standards should be further refined or simplified.

The in-plane frequency direction resolution specification of 0.40 mm in PI-RADSv2 and v2.1, while attainable, typically requires a compromise. For a field-of-view (FOV) of 16 cm, 400 readout samples are required, and this increases proportionately for larger FOVs. Compared to, for example, 320 samples, this either prolongs the echo duration, extending the entire multi-echo train, or requires an increased bandwidth to sample the requisite points more quickly, causing diminished signal-to-noise ratio (SNR) [13]. These observations led us to investigate whether somehow degrading the 0.40-mm frequency specification while making the 0.70 mm phase specification could result in improved performance.

In prostate MRI, image quality is a key factor, as poor image quality is known to adversely affect cancer detection rates [14, 15]. In our own practice, if an axial T2WI scan is deemed to have inadequate quality, typically due to motion artifact, then an additional T2WI scan is immediately performed using the PROPELLER sequence [16–18], which provides reduced sensitivity to motion but requires 50% increased acquisition time when compared to conventional T2WI. Analogous to this, the prostate imaging quality (PI-QUAL) version 1 [19] and version 2 [20] evaluation criteria have been established to define standards of adequate quality and also advise when a repeat scan is necessary.

Thus, the purpose of this work was to investigate whether a PI-RADS-adherent T2WI (PRT2-Adh) sequence could be substituted by a PI-RADS-modified T2WI (PRT2-Mod) sequence to provide equivalent in-plane spatial resolution, measured by area, while maintaining SNR and permitting reduced acquisition time. To assess possible clinical value, we hypothesized that the modified sequence would provide improved image quality with the potential for reduction of the need for rescans.

## Methods

### Ethics approval

**Fig. 1:**
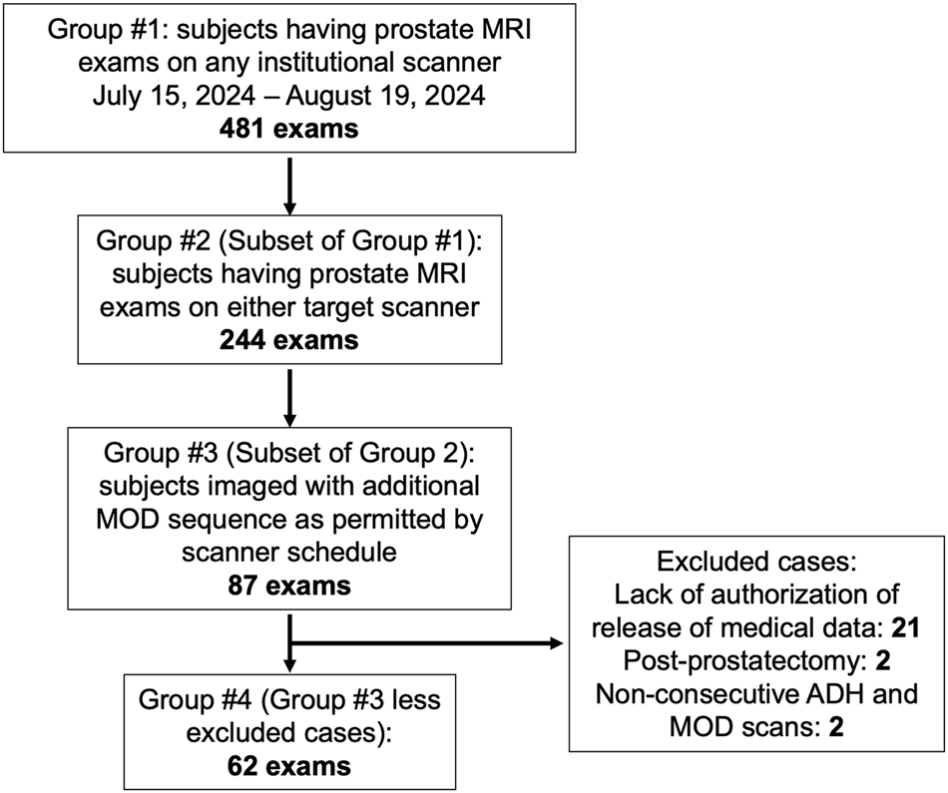
Flow of selection of subjects

Human studies were done under an Institutional Review Board-approved protocol (#15-000829), under which the requirement for written informed consent was waived. This work involved radiologist-authorized addition of a commercially available T2WI pulse sequence to the clinical MRI exam. All patients at our institution are given the option of authorizing that their anonymized medical data be made available for research, and all 62 participants did so. The flow of participant selection is shown in Fig. 1. Demographics of the participants are shown in Table 1.

**Table 1:** Demographics and axial T2WI scan parameters of 62 participants

Age range	48–83 years; median 67				
Weight range	64–138 kg; median 88.6				
BMI range	22.5–44.7 kg/m^2^; median 27.2				
PSA range	< 0.05 (undetectable) –21 ng/mL; median 5.5				
Prostate volume:	11–130 cc; median 49				
Total number of participants					62
Treatment-naive				54	
PI-RADS 2			24		
PI-RADS 3			10		
PI-RADS 4			10		
PI-RADS 5			10		
Post-treatment for prostate cancer				8	
Local cryoablation			4		
Chemo and hormonal therapy			1		
Chemo and ^a^ADT			1		
^a^ADT			1		
Bracytherapy			1		
Number of exams at each axial FOV					
16 cm				51	
18 cm				8	
20 cm				3	

Number of axial slices (min, max, and median): 28, 47, and 36

Acquisition time (PRT2-Adh) (min:s; min, max, and median): 2:57, 5:04, and 3:55

Acquisition time (PRT2-Mod) (min:s; min, max, and median): 2:16, 3:56, and 3:01

Reduction in acquisition time PRT2-Mod *versus* PRT2-Adh (s; min, max, and median): 38, 80, and 54

^a^ Androgen deprivation therapy

### MRI pulse sequences

MRI was performed at 3 T (Premier, GE Healthcare, Waukesha, WI, USA). Parameters of the reference PRT2-Adh fast-spin-echo sequence, routinely used in our practice, are shown in Table 2.

**Table 2:** Acquisition parameters for axial PI-RADS-adherent (PRT2-Adh) and modified (PRT2-Mod) T2-weighted spin-echo multislice of prostate

Parameter	PRT2-Adh	PRT2-Mod
Frequency × phase directions	A/P × L/R	A/P × L/R
^a^FOV (A/P × L/R)	16 × 16 cm^2^	16 × 16 cm^2^
Slice thickness	3 mm	3 mm
Slice-to-slice increment	3 mm (abutting)	3 mm (abutting)
In-plane sampling (frequency × phase)	400 × 230	320 × 280
In-plane resolution (frequency × phase)	0.40 × 0.70 mm^2^	0.50 × 0.57 mm^2^
In-plane resolution (area)	0.280 mm^2^	0.285 mm^2^
Pixel size (after interpolation)	0.156 × 0.156 mm^2^	0.156 × 0.156 mm^2^
Signal bandwidth	±64 kHz	±32 kHz
^b^Time between echo centers in readout	8.17 ms	10.38 ms
Echo train length (no. of echoes/repetition)	24	21
Sampling time per slice per repetition	217 ms	230 ms
Repetition time (TR) (15 slices per pass)	3,255 ms	3,450 ms
Effective echo time (TE)	150 ms	150 ms
Acceleration factor (ARC, along phase direction)	1.5	1.5
Number of averages	2	1
^c^No phase wrap (NPW)	Enabled	Enabled
Scan time per pass of 15 slices	46 s (14 repetitions)	69 s (20 repetitions)
Total scan time for 30 slices with averaging	3:02	2:18

*A/P* Anterior/posterior, *L/R* Left/right

^a^ Field of view is weight-based; the default is 16 cm for patients < 100 kg, 18 cm for 100–120 kg, and 20 cm for > 120 kg

^b^ Inter-echo time and thus total scan time can change slightly with oblique angulation

^c^ Enabling of NPW doubles the prescribed phase FOV and the number of prescribed phase encodings, but only the prescribed FOV is displayed

The sequence acquires abutting 3-mm thick slices in two passes to avoid slice-to-slice interference, one for the even-numbered and one for the odd-numbered slices., with 24 echoes sampled per repetition. Using a signal bandwidth of ±64 kHz, 400 points were sampled per echo, providing 0.40 mm frequency resolution for a 16-cm FOV. Two-hundred-thirty phase encodes provided 0.70-mm resolution in 16 cm; with an acceleration factor R of 1.5, a single image could be formed with data from 14 repetitions.

The vendor’s deep learning reconstruction [21–23] was performed at a level (“Medium”) as preferred by radiologist evaluation [24]. Two-fold averaging was done for an adequate SNR. In our clinical practice, the default FOV is 16 cm, but for patients weighing between 100 kg and 125 kg, the FOV is increased to 18 cm, while for those over 125 kg to 20 cm, without change in the 400 × 230 sampling. For such patients, the in-plane resolution is coarser and no longer PI-RADS minimum technical standards-adherent, but the intrinsic SNR is higher when compared to 16-cm FOV in proportion to the increased pixel size (26.5% higher for 18-cm FOV and 56% higher at 20-cm FOV). Repetition time, being the product of the sampling time per slice with the number of slices per pass, was 3,255 ms for the 15 slices/pass (30 total slices) assumed in Table 2. This results in an acquisition time of about 46 s per pass of odd or even slices, and with two-fold averaging per pass, a total of 182 s for the 30 slices.

Parameters of the modified T2WI sequence—PRT2-Mod—are also shown in Table 2. Many are the same as for the PRT2-Adh sequence, including 3-mm abutting slices, echo time 150 ms, deep learning “Medium” reconstruction, and acceleration factor R of 1.5. The principal change is that 320 samples were sampled per echo, providing 0.50-mm resolution for a 16-cm FOV, but with reduced, ±32 kHz *versus* ±64 kHz bandwidth. This slightly increases the echo-to-echo time (Table 2), limiting the readout train to an echo train length of 21. The √2 SNR restoration provided by the reduced bandwidth [13] allows a single acquisition, *i.e*., no averaging. To provide the same in-plane resolution by area as the PRT2-Adh sequence, 280 phase encodes were used, providing 0.57-mm phase resolution. This increases the number of repetitions required for a single pass to 20. Total scan time for two passes with one average was 138 s. For both the PRT2-Adh and PRT2-Mod acquisitions, each acquired image was interpolated using zero padding [25] to 1,024 × 1,024 samples, giving a pixel size of 0.156 × 0.156 mm^2^ for display.

### Phantom experiments

Experiments were done with phantoms to assess the SNR and in-plane spatial resolution of the two sequences. SNR was evaluated using a custom-built pelvic phantom, equivalent to a body mass index of 26 kg/m^2^ [26]. Thirty-two abutting 3-mm-thick axial slices were imaged using the PRT2-Adh and PRT2-Mod sequences of Table 1. Two scans were done for each, allowing test-retest. The signal S was measured at four levels in one of the scans for each sequence, taken as the average within a 2.5-cm diameter region of interest. Noise was taken at each level as the standard deviation σ in the same region-of-interest in the difference between the test-retest images [27]. SNR was taken as √2 (S/σ).

In-plane resolution was evaluated using replicates of a phantom with known spatial resolution placed within a water bath to allow simultaneous assessment of frequency and phase resolution [26], adapted to allow 0.6-mm and 0.5-mm measurement. It was imaged using 30-slice versions of both sequences with a FOV of 16 cm. As a reference, it was also imaged using the PRT2-Mod sequence with a FOV of 20 cm.

### Human studies

The PRT2-Mod sequence was incorporated into the clinical MRI protocol, schedule permitting, on two of the institutional MRI scanners on which prostate MRI exams are routinely performed. Sixty-two consecutive subjects were enrolled from July 15, 2024, to August 19, 2024. All subjects were men with suspected or known prostate cancer; 54 participants were treatment-naïve, and eight were evaluated post-therapy for prostate cancer, but none had prostatectomy (Table 1).

Immediately before scanning, all patients were administered 1 mg/mL glucagon (Glucagon Nova Plus, Fresenius Kabi) subcutaneously to arrest rectal peristalsis. Signal reception was done using anterior blanket and posterior in-table receiver coil arrays (15 elements each). For no participant was an endorectal coil used. After a localizer scan, sagittal T2WI sequences were acquired to align the slice select direction of the obliqued axial series with the centerline of the prostate. The number of axial slices was patient-specific, covering from the superior aspect of the seminal vesicles to the inferior aspect of the prostate apex. Repetition time was selected as the minimum allowable for the number of slices.

Once the obliquity and number of slices were determined, the axial PRT2-Adh sequence was run. This was immediately followed by the PRT2-Mod sequence using the same, patient-specific slice sampling and orientation. All 62 subjects were imaged with both sequences.

### Radiological evaluation

A total of 124 T2WI series were evaluated, two each (PRT2-Adh and PRT2-Mod) from each of 62 exams. Image series were evaluated by four radiologist readers (A.T.F., A.K., N.T., and J.T.), all of them with over 10 years’ experience in prostate MRI. The first 20 exams were evaluated by all four readers, and the subsequent 42 exams by exactly two readers, 21 per reader. Data for each exam were presented as a separate pseudo-exam comprised solely of the two (PRT2-Adh and PRT2-Mod) axial series, with the order randomized and all sequence- and patient-related information removed. The signal ranges for each series were normalized to permit the same window and level for viewing the two series simultaneously. Images were viewed using Visage 7 client software (Visage Imaging).

For each exam, the image quality of the two sequences was individually evaluated. Two sets of evaluation criteria were used. The first was a four-point (0–3) scale for diagnostic quality (DQ) developed and used by our reviewers previously [24, 28] prior to the definition of PI-QUALv2 [20]. The scale is defined as follows:

- DQ 0 = obviously non-diagnostic, severely limited by markedly poor SNR, gross motion or other artifact, or otherwise non-interpretable;
- DQ 1 = marginally non-diagnostic, with SNR, resolution, or artifact limiting interpretation, generally requiring a rescan;
- DQ 2 = non-ideal in quality, such as due to slight blurring, but still allowing interpretation;
- DQ 3 = high DQ.

Evaluations were based on SNR, sharpness, and the level of any artifact interfering with an assessment of the prostate. For inter-reader calibration, prior to the evaluation, all readers were presented with four exams at each DQ score, not part of the study, scored in consensus [28].

Each sequence was also evaluated for image quality using the PI-QUALv2 criteria [20] for axial T2WI. For all exams, both sequences met the essential requirement for 3 mm-thick axial slices. The three other criteria (SNR, delineation, and artifact) for the axial series were each evaluated as adequate or not using a Y/N (1/0) scale. For each sequence, results for each individual criterion, as well as the sum (integer scores 0–3), were tabulated. For calibration, prior to review, all readers were provided images from the original work [20], which showed sample Y/N scores.

The two image series (A, B) for each exam were also evaluated for preference using a five-point scale:

-2 = strongly prefer series A;
-1 = prefer series A;
- 0 = prefer neither series;
- +1: prefer series B;
- +2: strongly prefer Series B.

For each exam, the PRT2-Adh and PRT2-Mod series could be visualized side by side, but the order (A, B) was randomized, and all descriptive annotations were removed.

### Statistics

Each score was averaged across all reviewers for that exam. The differences in DQ score, PI-QUALv2 individual scores and sum, and reader preference from the null result were evaluated using the Wilcoxon signed-rank test [29] with significance taken as *p* < 0.050. Inter-reader consistency was evaluated using Cohen’s κ [30] and Krippendorf’s α [31]. We acknowledge that the DQ scores and summed PI-QUAL scores are subjective and somewhat redundant with each other. Because these are both ranked, noncardinal variables, we assessed their correlation with 95% confidence intervals (CI) using Spearman’s coefficient [29] using SciPy [32] with bootstrapping.

Acquisition times for the PRT2-Adh and PRT2-Mod sequences were taken from exam DICOM header data and plotted *versus* the number of acquired slices, and the average time per slice was determined by standard regression analysis.

## Results

### Phantom measurements

**Fig. 2:**
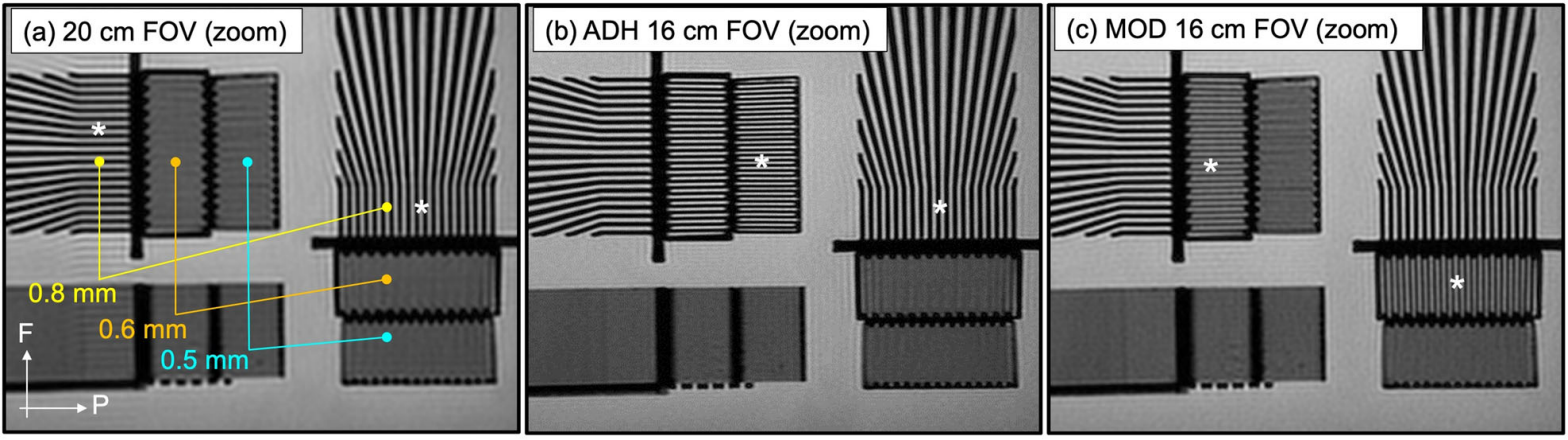
Images of the spatial resolution phantom. **a** Zoomed axial image acquired at 20 cm FOV shows the frequency (F) and phase (P) directions and quantifies specific resolution patterns. The limiting resolution pattern is identified in each direction with an asterisk (*), 0.8 mm in both F and P directions. **b** Zoomed axial image acquired at 16 cm FOV using the PI-RADS-adherent (PRT2-Adh) sequence. The 0.5 mm pattern is resolved in the frequency direction and the 0.8 mm pattern in the phase direction. **c** Zoomed axial image acquired at 16 cm FOV using the modified (PRT2-Mod) sequence. The 0.6 mm pattern is resolved in the frequency direction, inferior to the PRT2-Adh result, and the 0.6 mm pattern is resolved in the phase direction, superior to the PRT2-Adh result. In both (**b**, **c**), the resolution limits are coarser than the 0.156 × 0.156 mm^2^ interpolated size used for display

The mean ratio of SNRs in the PRT2-Mod *versus* PRT2-Adh sequences in the phantom was 1.04 ± 0.06; *i.e*., the SNRs were not distinguishable. Images of the resolution phantom are shown in Fig. 2. Resolvable patterns are consistent with the in-plane sampling and resolution of Table 2, illustrating the tradeoff in resolution.

### Demographics and scan time summary

**Fig. 3:**
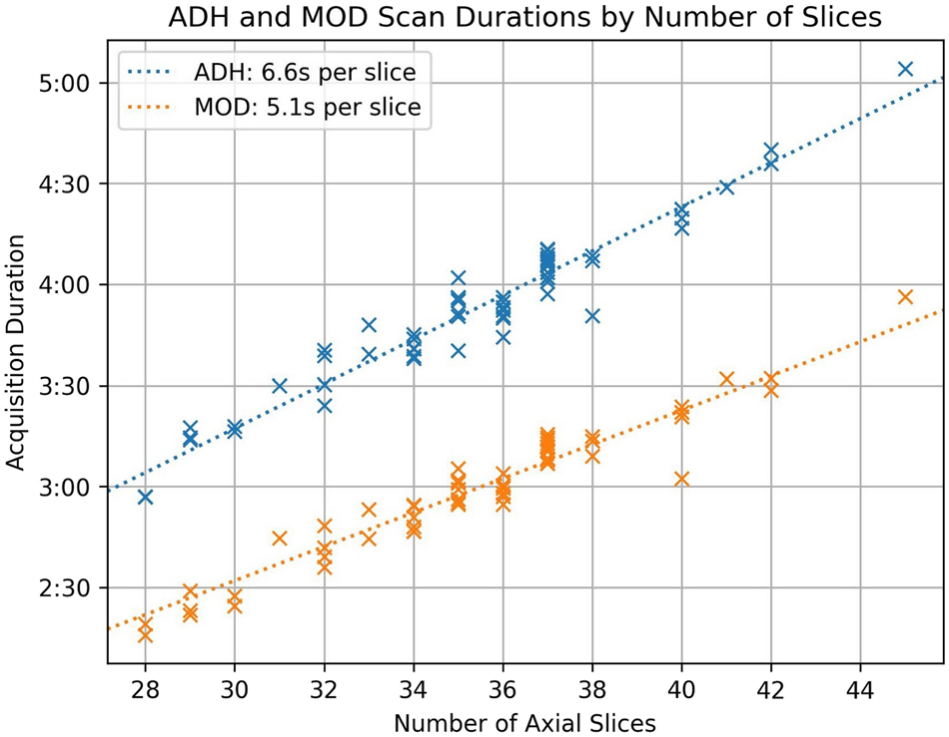
Plot of acquisition times of the PI-RADS-adherent (PRT2-Adh) and modified (PRT2-Mod) sequences *versus* the number of axial slices used. Times were taken from the exam DICOM headers. For a given slice number, the times vary due to subtle changes in the timing of the echo train due to differences in angulation subject-to-subject, and these, in turn, can vary for the PRT2-Adh and PRT2-Mod sequences. Lines of regression show the mean acquisition times per acquired slice, 6.6 s/slice for PRT2-Adh and 5.1 s/slice for PRT2-Mod. The difference, 1.5 s/slice, is a 23% reduction in acquisition time per slice provided by the PRT2-Mod *versus* PRT2-Adh sequence

In addition to demographics, Table 1 shows the range of various T2WI parameter values across the 62 participants. Median acquisition times were 3:55 min:s for the PRT2-Adh and 3:01 min for the PRT2-Mod sequences, respectively, with a median reduction of 54 s provided by PRT2-Mod. Figure 3 shows acquisition times for the two sequences *versus* the number of acquired axial slices. The reduction in acquisition time provided by PRT2-Mod *versus* PRT2-Adh is 1.5 s/slice, 23%. Supplemental Fig. S1 shows the axial FOV used as a function of patient weight.

### DQ scores

**Fig. 4:**
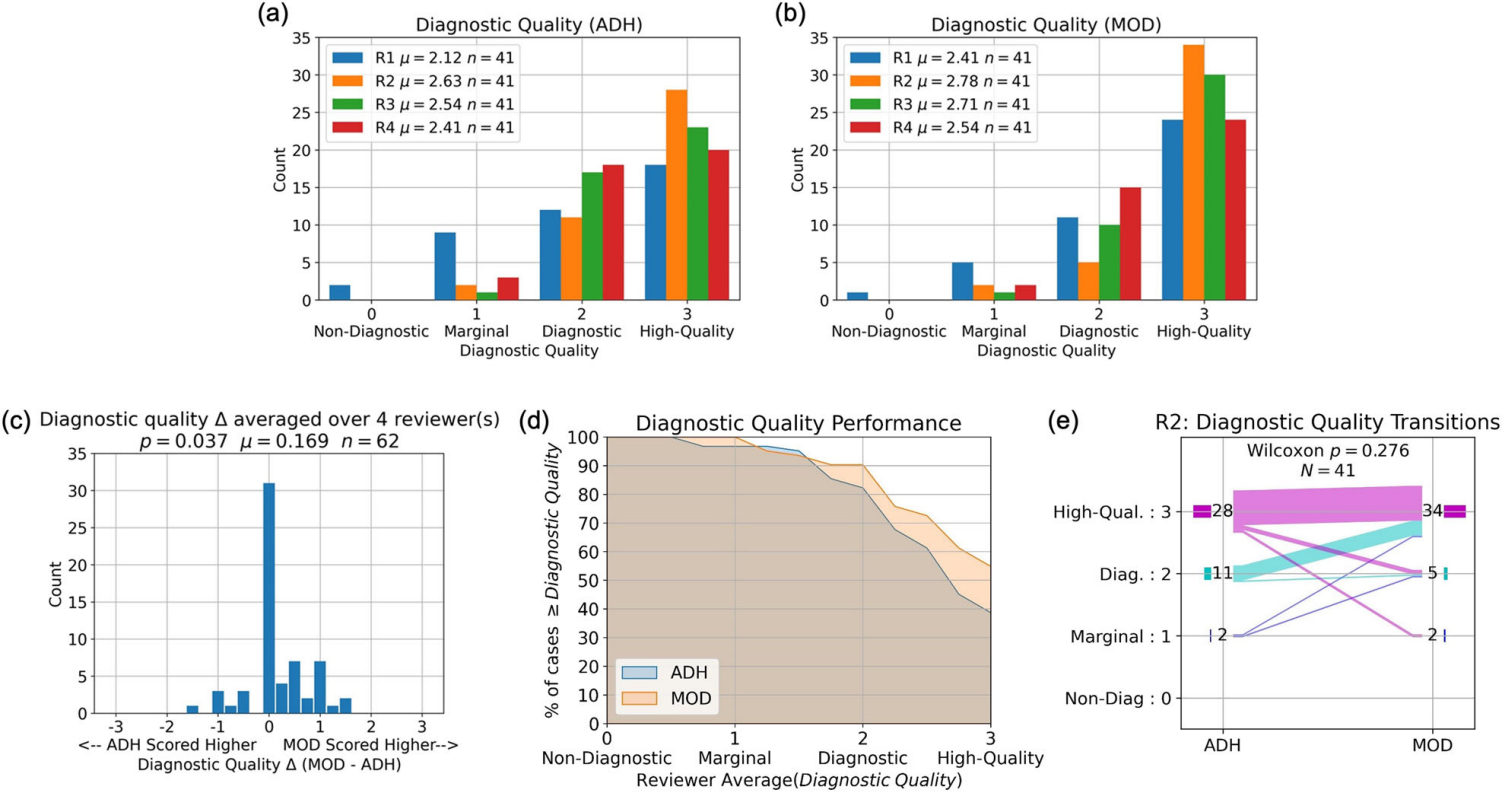
DQ results. Color-coded histograms of scores of the individual readers, R1–R4, are shown for the PI-RADS-adherent (PRT2-Adh) sequence (**a**) and modified (PRT2-Mod) sequence (**b**). Means values (µ) are also indicated. **c** Histogram of the difference in reader-averaged DQ scores (mean value µ = 0.169). The distribution shows statistically significant superiority of the DQ provided by the PRT2-Mod *versus* PRT2-Adh sequence (*p* < 0.037). **d** Plot of the performance of the PRT2-Adh and PRT2-Mod sequences, showing the percentage of exams whose DQ value matches or exceeds the abscissa value. **e** Histograms from Reader R2 illustrating the typical distribution of scores. The scores for each exam are connected by a line. For many exams, the PRT2-Adh and PRT2-Mod sequences were evaluated as providing high quality. For exams with different PRT2-Adh and PRT2-Mod scores, the trend was for the PRT2-Mod sequence to be evaluated more favorably

Figure 4 shows results for DQ, with a trend for more favorable scores for the PRT2-Mod sequence for all readers. Figure 4c shows the difference in reader-averaged DQ scores across the 62 exams. The 0.25 abscissa sampling is due to the averaging of up to four integer reader scores. The superiority of the PRT2-Mod sequence compared to PRT2-Adh resulted to be significant (*p* < 0.037). Figure 4d is a “performance plot” showing how, at virtually all DQ values ≥ 0.5, the PRT2-Mod sequence is superior. Figure 4e shows the frequencies of individual DQ scores for reader R2: scores for many exams are identical, but exams with different scores tend to favor the PRT2-Mod sequence.

The Krippendorf α for the four reviewers in evaluating all 124 series for DQ was 0.64 (substantial agreement [33]). Supplemental Fig. S2 shows inter-reader agreement matrices and Cohen’s κ values for the six inter-reader comparisons.

### PI-QUAL-v2 scores and reader preference

**Fig. 5:**
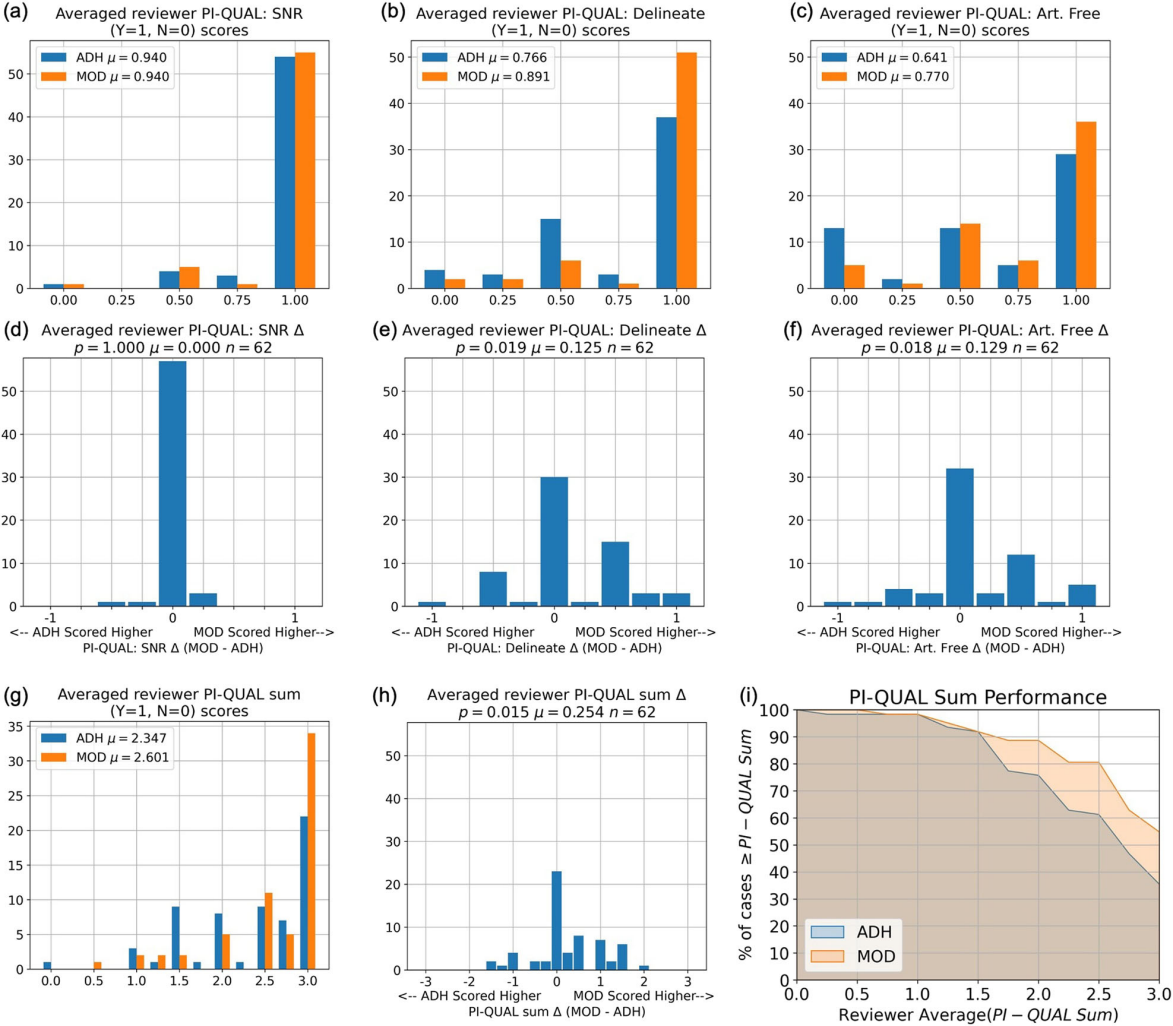
PI-QUALv2 results. **a** Histograms of the reader-averaged scores of the individual PI-QUAL criteria for SNR (**a**), delineation (**b**), and artifact-free (**c**). In all cases, there are 62 scores each for PRT2-Mod and PRT2-Adh, and the mean values (µ) are indicated. Histograms of the difference between reader-averaged scores of PRT2-Mod *versus* PRT2-Adh for the individual PI-QUAL criteria for SNR (**d**), delineate (**e**), and artifact-free (**f**), with statistical significance (*p*) *versus* equivalent performance indicated. **g** Histogram of the reader-averaged sums of the PI-QUALv2 scores for SNR, delineation, and artifact (sum = 0–3). **h** Histogram of the difference in reader-averaged summed PI-QUAL scores. **i** Plot of performance of PRT2-Adh and PRT2-Mod sequences as a function of PI-QUALv2 scores

Figure 5 shows results of the PI-QUAL evaluation, with results for the three individual criteria in (a–f) and for the summed scores in (g, h). The superiority of the PRT2-Mod sequence was significant in delineation (e), artifact-free (f), and the overall sum (h). Figure 5i is the performance plot using PI-QUAL sum scores analogous to Fig. 4d.

The Krippendorf α for the PI-QUALv2 evaluation was 0.49, showing moderate agreement [33]. Supplemental Figure S3 shows inter-reader comparisons. The Spearman coefficient comparing reader-averaged DQ scores (0–3) with reader-averaged PI-QUAL scores (0–3) across all 124 series was 0.83 (CI 0.74, 0.90), indicating a very high correlation.

**Fig. 6:**
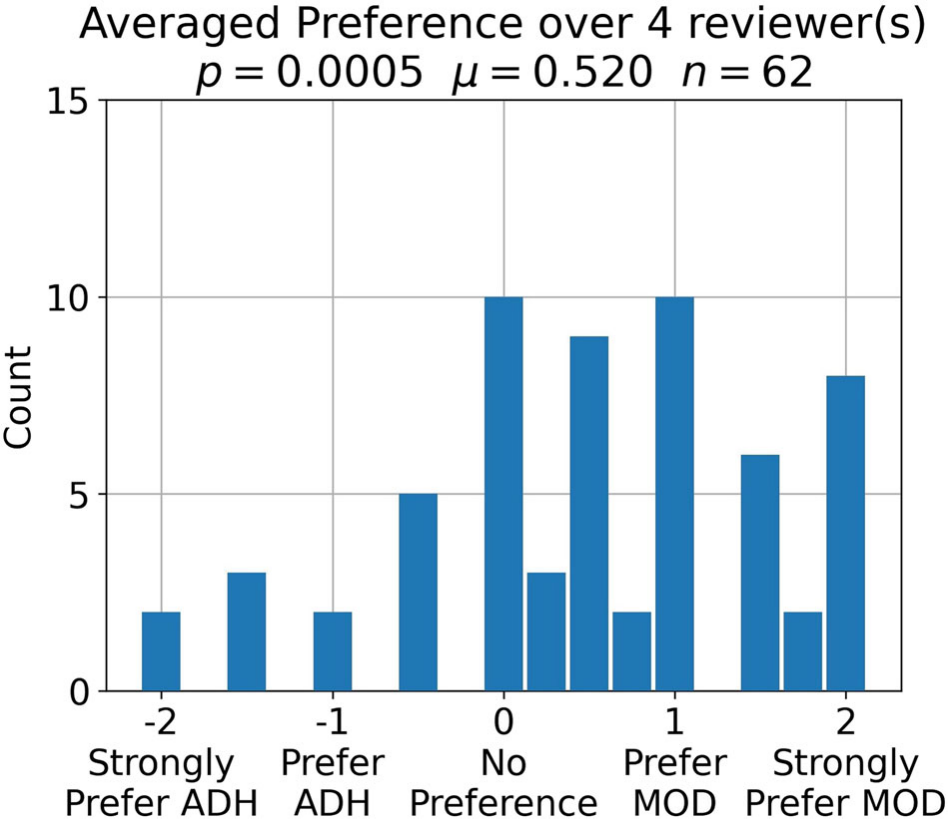
Plot of histogram of reader-averaged preference scores (mean value µ = 0.520). The distribution shows statistically significant superior reader preference for the PRT2-Mod *versus* PRT2-Adh sequence (*p* < 0.0005). Forty of the 62 scores were positive, showing a preference for PRT2-Mod

Figure 6 shows that reader preference for the PRT2-Mod sequence is significant (*p* < 0.001). The PRT2-Mod sequence was preferred in 40 of 62 exams. The Krippendorf α was 0.60, showing moderate-to-substantial agreement [33]. Supplemental Fig. S4 shows inter-reader comparisons.

**Fig. 7:**
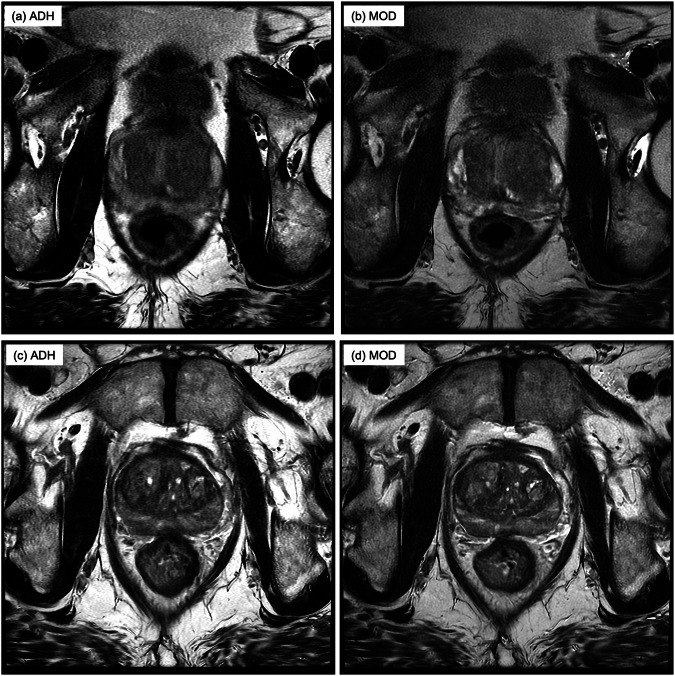
Examples of improvement in DQ provided by PRT2-Mod *versus* PRT2-Adh sequence. **a**, **b** Side-by-side comparison of full 16 cm FOV axial T2WI images acquired using the PRT2-Adh (**a**) and PRT2-Mod (**b**) sequences from Participant 45 (93.5 kg). In this example, the average DQ score of the two readers of the PRT2-Adh series was 0.5, requiring rescan, while that of the PRT2-Mod series was 2.0, adequate for diagnosis. **c**, **d** Side-by-side comparison of 16 cm FOV images acquired using the PRT2-Adh (**c**) and PRT2-Mod (**d**) sequences from Participant 18 (78 kg). PRT2-Adh series was evaluated by the four readers as DQ 2.25, but the PRT2-Mod series was evaluated as 3. Supplemental Videos 1 and 2 compare the full series from these two examples

Figure 7 shows representative image results. Videos corresponding to the two exams in the figure are presented in Supplemental Videos S1 and S2.

## Discussion

We have shown how the in-plane resolution of a PI-RADS-adherent T2WI spin-echo sequence for prostate MRI can be modified from the minimum standard 0.40 mm frequency × 0.70 mm phase (0.280 mm^2^ pixel area) to a more isotropic 0.50 mm frequency × 0.57 mm phase resolution with essentially identical resolution area (0.285 mm^2^) and provide a median reduction of 54 s of acquisition time, as well as significantly improved DQ, PI-QUAL score, and reader preference *versus* the reference PRT2-Adh sequence.

The technical basis for the reduction in acquisition time was to note that high frequency resolution typically requires either a long duration of each echo and thus extension of the echo train in a multi-echo sequence or a high bandwidth to allow rapid sampling of the many required frequency points. In this work, reducing the number of samples in the frequency direction allowed two-fold bandwidth reduction, providing SNR restoration of √2 or 40%, enabling only 1× *versus* 2× averaging. The reduced frequency resolution was then compensated with increased phase sampling, giving equivalent in-plane resolution area to the PRT2-Adh sequence, equivalent SNR, but still allowing net reduced acquisition time.

Other than targeting high in-plane spatial resolution, the motivation for the intentionally anisotropic 0.40 mm frequency × 0.70 mm phase resolution standard in PI-RADS is not clear. It does not seem to be anatomically based, as the frequency direction (left-to-right, anterior-to-posterior) is not specified. This current work suggests that defining an in-plane resolution target measured by area and allowing flexibility in meeting this may provide technical advantages. In fact, PI-RADSv1 [1] alludes to T2-WI in-plane resolution in such a general manner.

The DQ scores showed that both the PRT2-Adh and PRT2-Mod sequences provided high quality in a large percentage of the exams. However, the PRT2-Mod was significantly superior (Fig. 4c).

### PI-QUAL scores

The PI-QUAL evaluation showed that the SNRs in the PRT2-Adh and PRT2-Mod sequences were comparable and adequate in most cases (Fig. 5a). In only five of the 62 exams were the mean SNR scores different between the two sequences. Only one exam received an average SNR score of 0, assigned to both the PRT2-Adh and PRT2-Mod sequences. This was because the 130-kg subject was erroneously imaged with 16 cm, rather than the protocol-specified 20 cm FOV. This suggests that the weight-based FOV consistently guarantees adequate SNR and, in effect, removes SNR from being a variable of concern.

Although the PRT2-Adh sequence was adequate for delineation of the prostate in that 55/62 (88.7%) of the cases had average scores ≥ 0.5 (Fig. 5b), the PRT2-Mod sequence had statistically superior performance (Fig. 5e). Similarly, the PRT2-Mod sequence had statistically superior performance *versus* PRT2-Adh for the third PI-QUAL criterion, artifact-free (Fig. 5f). Consequently, the PRT2-Mod sequence was also superior to PRT2-Adh in the overall PI-QUAL sum score (Fig. 5g).

Figures 4e and 5i are the performance plots generated using the DQ and PI-QUAL scores, respectively. Not surprisingly, because these two evaluation criteria have some level of redundancy, the plots show similar trends. At moderate to high scores (> 2 for both plots), the PRT2-Mod performance exceeds that of PRT2-Adh, typically by ten percentage points or more. For scores in this range, because the image quality is high for both, there might be little significant difference in diagnostic performance. An example is the comparison in Fig. 7c, d. On the other hand, for image quality scores which are marginal (< 2 in both plots), the small but nonetheless superior performance of the PRT2-Mod *versus* PRT2-Adh sequence can potentially make a difference in not requiring a rescan. An example is given in Fig. 7a, b.

It is instructive to look at the 13 exams in which one sequence or the other had a mean DQ score of 1.5 or less, having marginal or poor DQ. In only 2 of 13 exams did both PRT2-Adh and PRT2-Mod sequences have poor quality. One of these was the above-described erroneous FOV prescription, and a second was degraded by motion in both the PRT2-Adh and PRT2-Mod results. In the remaining 11 exams, only one of the other sequences was non-diagnostic. That is, in all but 2 of the 62 cases, one or both T2-WI sequences had DQ. Given the equivalent, high SNR scores and generally high delineation scores for both sequences, we infer that the superiority of the PRT2-Mod sequence was primarily due to reduced likelihood of artifacts due to reduced acquisition time.

We note that for the simple head-to-head blinded comparison used for reader preference, the PRT2-Mod sequence was preferred with a greater degree of significance (*p* < 0.0005) than either the DQ score or PI-QUAL criteria.

Reduced acquisition time may favorably affect workflow. In addition to the reduction of the axial T2WI scan by about 1 min, incorporation of this PRT2-Adh sequence into the sagittal or coronal T2WI scan can result in additional, similar timesaving. Perhaps more importantly, if the PRT2-Mod sequence in general provides for 90–95% of Axial T2WI scans to have diagnostic image quality *versus* 85–90% with the PRT2-Adh sequence, as suggested in the performance plots, this will result in reduction of rescans which in our practice each requires close to five min of scan time.

The PRT2-Adh reference sequence already used an acceleration factor *R* = 1.5, as well as benefited from the reduction in acquisition time allowed by the noise reduction intrinsic to the deep learning reconstruction, estimated as 33% in [23]. As shown, the changes made in defining the PRT2-Mod sequence provided an additional 26% reduction in acquisition time, similar to those attained with acceleration and deep learning, as well as improved image quality.

This study has limitations. It was implemented by modifying a specific PI-RADS-adherent T2-WI sequence on two identical scanners of one vendor at one institution, and thus, the degree of generalizability is not clear. Starting with a different but still adherent sequence on the same vendor, *e.g*., by Lee et al [23], may well provide a different degree of improvement. Sequence modification of a PI-RADS-adherent sequence on platforms of different vendors may involve other sequence variables than those described here. In addition, the number of exams was limited. It did not allow, for example, stratification of performance for specific subgroups such as treated *versus* non-treated subjects or low *versus* high body mass index subjects. However, this is often a limitation of any newly described method. We note that the PRT2-Mod sequence matches the PRT2-Adh sequence in SNR, in in-plane resolution by area, and it uses the same FOV and slice count as PRT2-Adh for all subjects.

In summary, we have shown that modification of a PI-RADS-adherent T2-WI sequence to allow coarser frequency resolution than the standard 0.40 mm but retaining equivalent 0.28 mm^2^ resolution area allows a median 54-s reduced acquisition time with significantly improved DQ, PI-QUAL score, and reader preference.

## Supplementary information

**Additional file 1: Supplemental Fig. S1.** Plot of FOV of T2WI sequence *versus* weight of patient. **Supplemental Fig S2**. Inter-reader agreement matrices and Cohen κ values for Diagnostic Quality. **Supplemental Fig. S3**. Inter-reader agreement matrices and Cohen κ values for summed PI-QUALv2 scores. **Supplemental Fig. S4**. Inter-reader agreement matrices and Cohen κ values for sequence preference. **Supplemental Video 1**. Side-by-side comparison of zoomed images from 32 slices from Participant #45 acquired using PRT2-Adh sequence (Left) in acquisition time 3:40 and PRT2-Mod sequence (Right) in time 2:42. See also Fig. 7a, b. **Supplemental Video 2**. Side-by-side comparison of zoomed images from 35 slices from Participant #18 acquired using PRT2-Adh sequence (Left) in acquisition time 3:56 and PRT2-Mod sequence (Right) in time 2:55. See also Fig. 7c, d.

Supplemental Video 1.

Supplemental Video 2.

## Data Availability

The datasets generated and analyzed during the current study are not publicly available but are available from the corresponding author on reasonable request.

## Abbreviations

DQ: Diagnostic quality
FOV: Field-of-view
PI-QUAL: Prostate imaging quality
PI-RADS: Prostate imaging and reporting data system
PRT2-Adh: PI-RADS-adherent T2WI
PRT2-Mod: PI-RADS-modified T2WI
SNR: Signal-to-Noise Ratio
T2WI: T2-weighted imaging
