# Supplementary material for: Improved image quality and reduced acquisition time in prostate T2-weighted spin-echo MRI using a modified PI-RADS-adherent sequence
Source: Eur Radiol Exp. 2025 May 24;9:55. doi: 10.1186/s41747-025-00595-w (PMC12103434; doi:10.1186/s41747-025-00595-w)
Supplement: Supplementary file 1 — Additional file 1: Supplemental Fig. S1. Plot of FOV of T2WI sequence versus weight of patient. Supplemental Fig S2. Inter-reader agreement matrices and Cohen κ values for Diagnostic Quality. Supplemental Fig. S3. Inter-reader agreement matrices and Cohen κ values for summed PI-QUALv2 scores. Supplemental Fig. S4. Inter-reader agreement matrices and Cohen κ values for sequence preference. Supplemental Video 1. Side-by-side comparison of zoomed images from 32 slices from Participant #45 acquired using PRT2-Adh sequence (Left) in acquisition time 3:40 and PRT2-Mod sequence (Right) in time 2:42. See also Fig. 7a, b. Supplemental Video 2. Side-by-side comparison of zoomed images from 35 slices from Participant #18 acquired using PRT2-Adh sequence (Left) in acquisition time 3:56 and PRT2-Mod sequence (Right) in time 2:55. See also Fig. 7c, d. [file 41747_2025_595_MOESM1_ESM.pdf]

Improved image quality and reduced acquisition time in prostate T2-weighted spin-echo MRI using a modified PI-RADS-adherent sequence

ELECTRONIC SUPPLEMENTARY MATERIAL

Eur Radiol Exp (2025) Riederer SJ, Borisch EA, Foremmingl AT, et al.

Supplemental Fig. S1. Plot of FOV of T2WI sequence *versus* weight of patient.

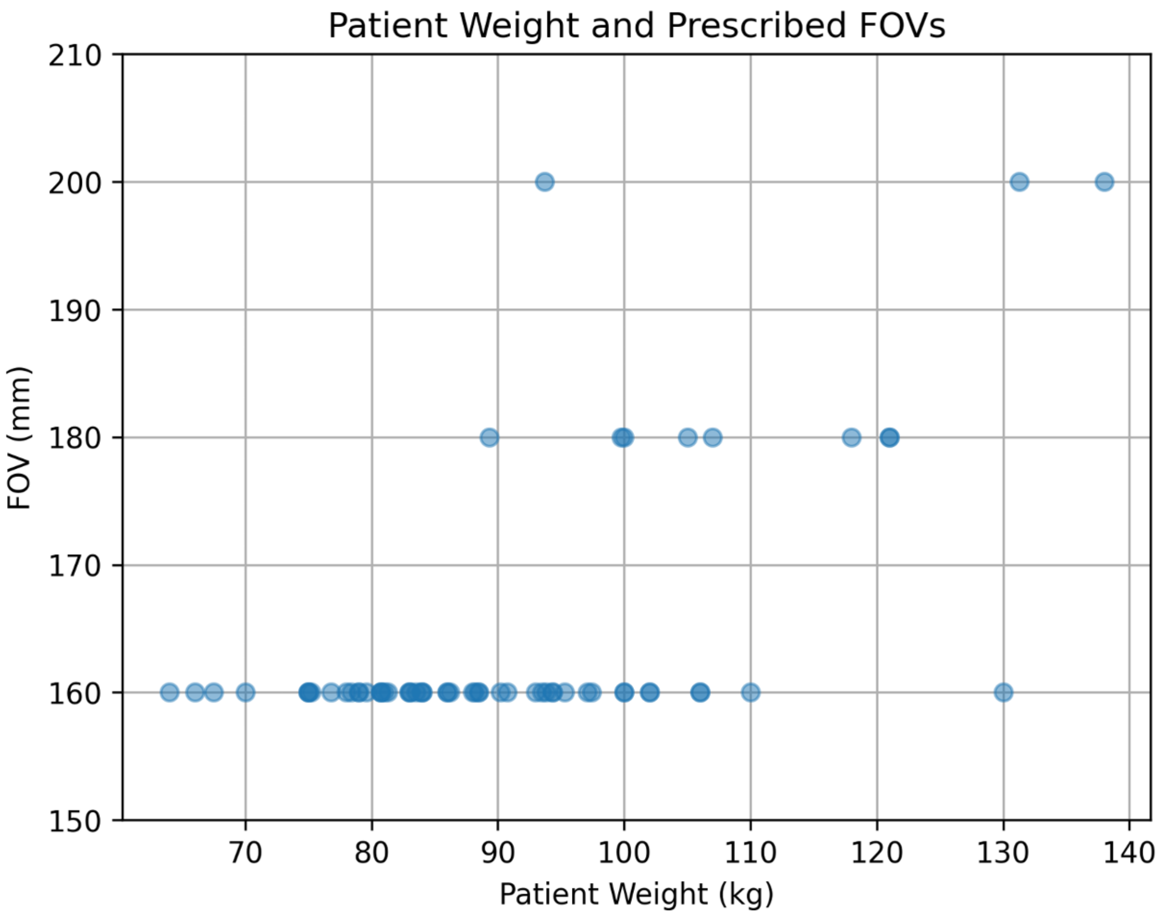

Supplemental Fig S2. Inter-reader agreement matrices and Cohen κ values for Diagnostic Quality

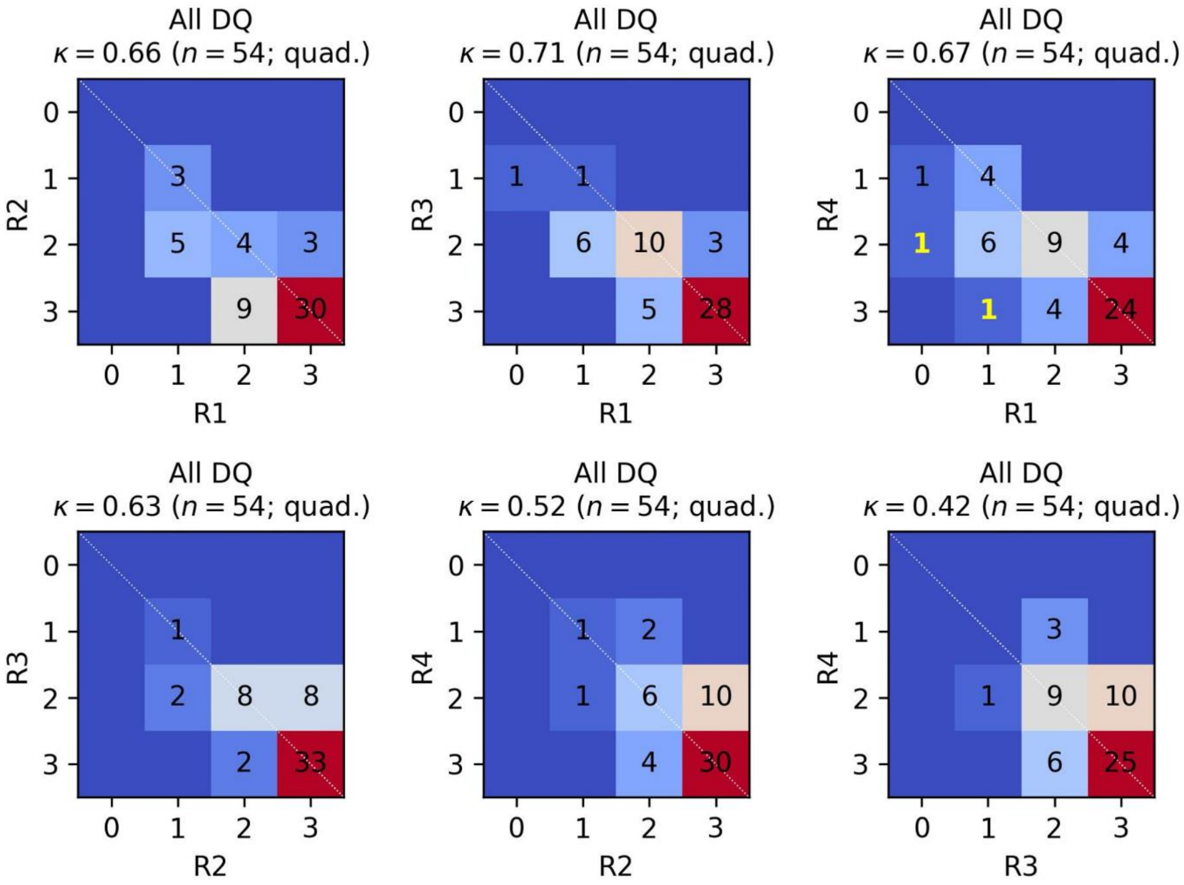

Supplemental Fig. S3. Inter-reader agreement matrices and Cohen κ values for summed PI-QUALv2 scores.

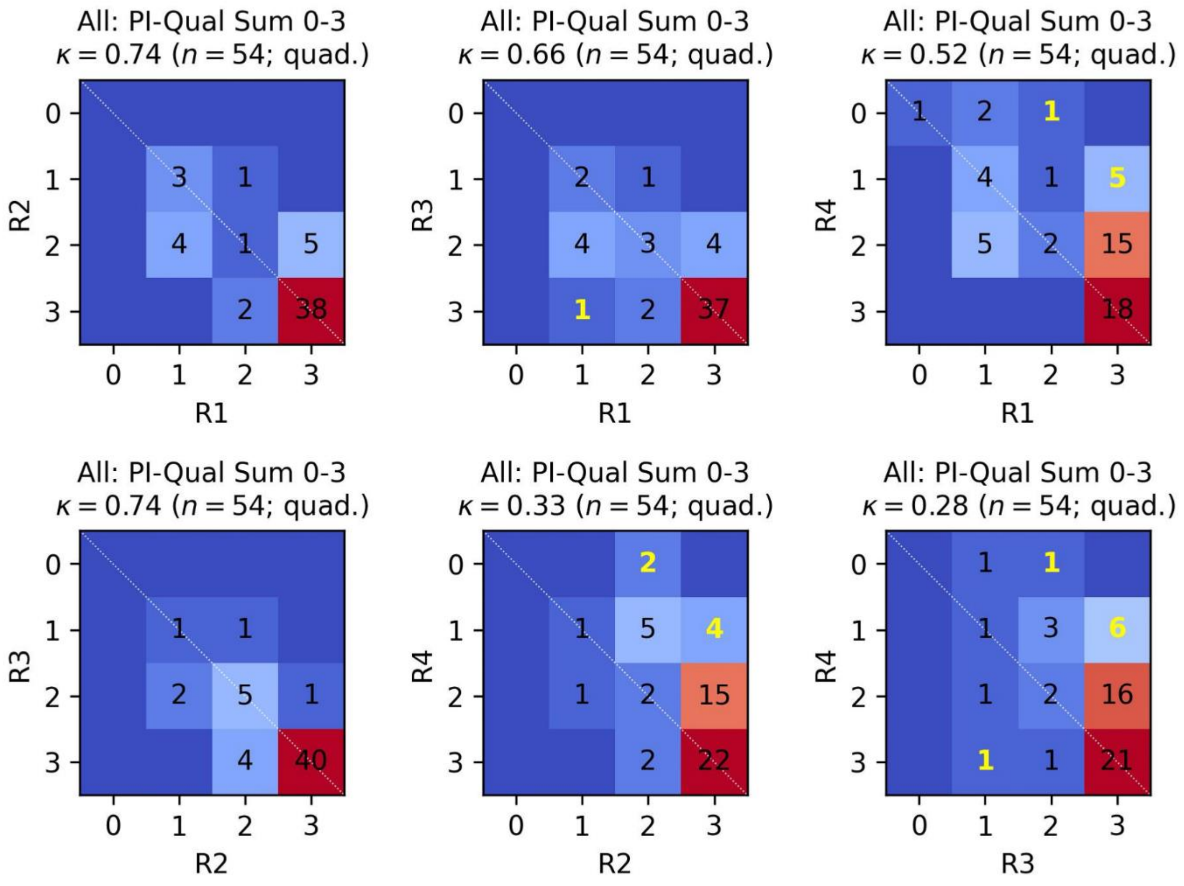

Supplemental Fig. S4. Inter-reader agreement matrices and Cohen κ values for sequence preference

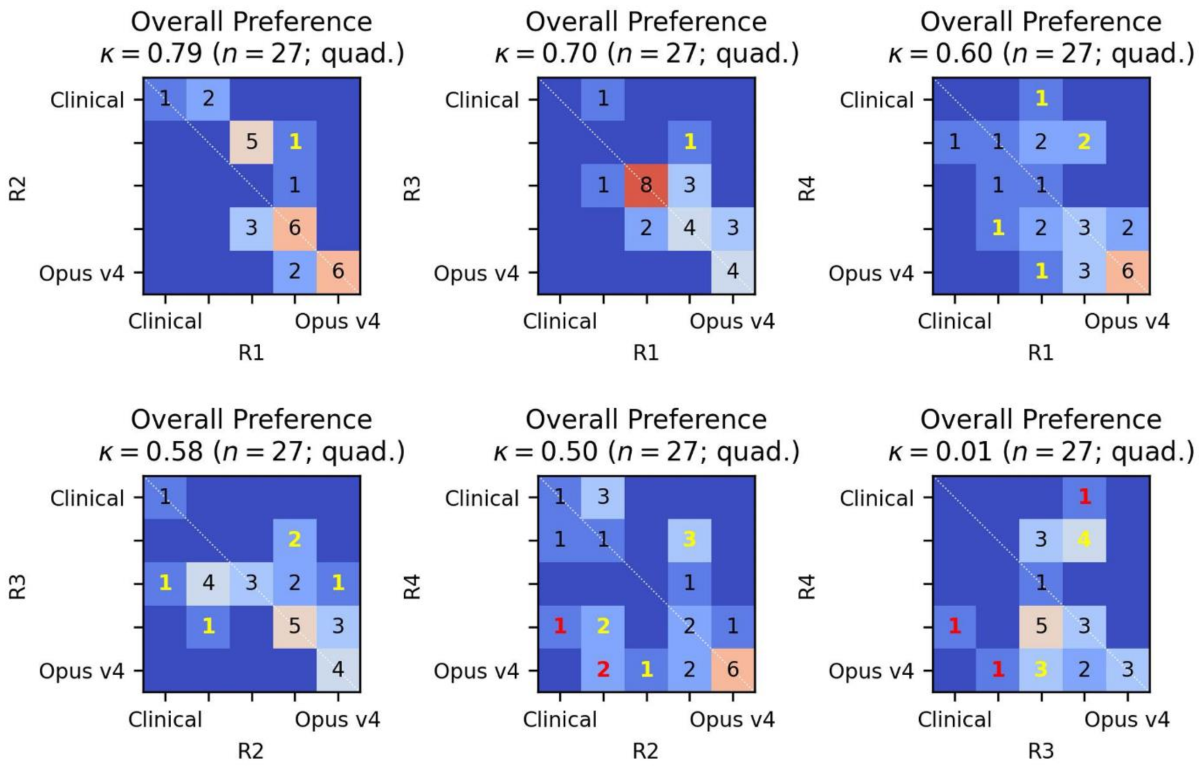

Supplemental Video 1. Side-by-side comparison of zoomed images from 32 slices from Participant #45 acquired using PRT2-Adh sequence (Left) in acquisition time 3:40 and PRT2-Mod sequence (Right) in time 2:42. See also Fig. 7a-b.

Supplemental Video 2. Side-by-side comparison of zoomed images from 35 slices from Participant #18 acquired using PRT2-Adh sequence (Left) in acquisition time 3:56 and PRT2-Mod sequence (Right) in time 2:55. See also Fig. 7c-d.
